# NK cell upraise in the dark world of cancer stem cells

**DOI:** 10.1186/s12935-021-02400-1

**Published:** 2021-12-19

**Authors:** Alireza Shokouhifar, Javad Firouzi, Masoumeh Nouri, Gholamreza Anani Sarab, Marzieh Ebrahimi

**Affiliations:** 1 Department of Molecular Medicine, Genomic Research Center, Birjand University of Medical Sciences, Birjand, Iran; 2 Cellular & Molecular Research Center, Birjand University of Medical Sciences, Birjand, Iran; 3 Department of Stem Cells and Developmental Biology, Cell Science Research Center, Royan Institute for Stem Cell Biology and Technology, ACECR, 16635-148 Tehran, Iran; 4 Department of Tissue Engineering & Regenerative Medicine, Iran University of Medical Sciences, Tehran, Iran; 5 R&D Department, Royan Stem Cell Technology Co., Tehran, Iran; 6 Department of Regenerative Medicine, Cell Science Research Centre, Royan Institute for Stem Cell Biology and Technology, ACECR, 14155-4364 Tehran, Iran

**Keywords:** NK cell, CSC, Immune cell-based therapy, CAR

## Abstract

One of the obstacles in treating different cancers, especially solid tumors, is cancer stem cells (CSCs) with their ability in resistance to chemo/radio therapy. The efforts for finding advanced treatments to overcome these cells have led to the emergence of advanced immune cell-based therapy (AICBT). Today, NK cells have become the center of attention since they have been proved to show an appropriate cytotoxicity against different cancer types as well as the capability of detecting and killing CSCs. Attempts for reaching an off-the-shelf source of NK cells have been made and resulted in the emergence of chimeric antigen receptor natural killer cells (CAR-NK cells). The CAR technology has then been used for generating more cytotoxic and efficient NK cells, which has increased the hope for cancer treatment. Since utilizing this advanced technology to target CSCs have been published in few studies, the present study has focused on discussing the characteristics of CSCs, which are detected and targeted by NK cells, the advantages and restrictions of using CAR-NK cells in CSCs treatment and the probable challenges in this process.

## Dormant stem cells hidden inside tumors

Cancer is one of the major mortality causes worldwide, accounting for about 20% of deaths in the developed countries annually [1]. Today, according to numerous clinical and preclinical studies, there are several available diagnostic and therapeutic methods that play crucial roles in the therapeutic and preventive processes [2]. Common therapies including surgery, radiotherapy, and chemotherapy have low degrees of efficacy in the treatment and also relief from recurrence [3]. One of the main reasons for cancer progression, metastasis and its treatment failure is tumor cells heterogeneity and the dormant cells with special ability of tumor development. These dormant cells, called cancer stem cells (CSCs), [4] are characterized by self-renewal, proliferation, differentiation into multiple cell types and drug resistance potential and are considered as the sparks of primary tumor cells [5]. Based on the evaluation of stemness characteristics and specific surface markers, CSCs can be distinguished from among the other tumor cells by identification methods listed in Table 1. Single-cell sequencing is one of the powerful tools for identifying CSCs from among other cells. Single-cell sequencing including single-cell transcriptome, epigenome, and genome sequencing technologies is used for characterizing the omic-scale features of heterogeneous cell populations such as stem cells. Many studies have demonstrated that hematopoietic stem cells, pre-leukemic stem cells and leukemic stem cells can be distinguished through combining single-cell transcriptomics such as transcriptome and genome (G&T-seq), transcriptome and DNA methylome (scM&T-seq), or genome, DNA methylome and transcriptome (scTrio-seq) [6, 7].

**Table 1:** Characterization of cancer stem cells

	Method		Procedure
Isolation and identification of CSCs	Side population detection		Sorting based on Hoechst dye efflux
	Cell surface markers detection		Sorting based on cell surface marker expression
	Culture of non-adherent		Sphere culture
Properties and characterization of CSCs	Tumorgenicity assay		Implantation of a single CSC for generating the entire tumor in a mouse model
	Self-renewal	Serial transplantation (single cell)	The low numbers of CSCs isolated from any generation of tumor should be able to give rise to a subsequent tumor in vivo
		In vitro renewal	Measuring the ability to form colonies through multiple generations in vitro
	Establishment of tumor heterogeneity		Determination of CSC-derived tumor heterogeneity by flowcytometry (surface markers) or immunohistochemistry

^a^The surface markers used based on tumor differentiation to identify CSCs are depicted in Table 2

**Fig. 1:**
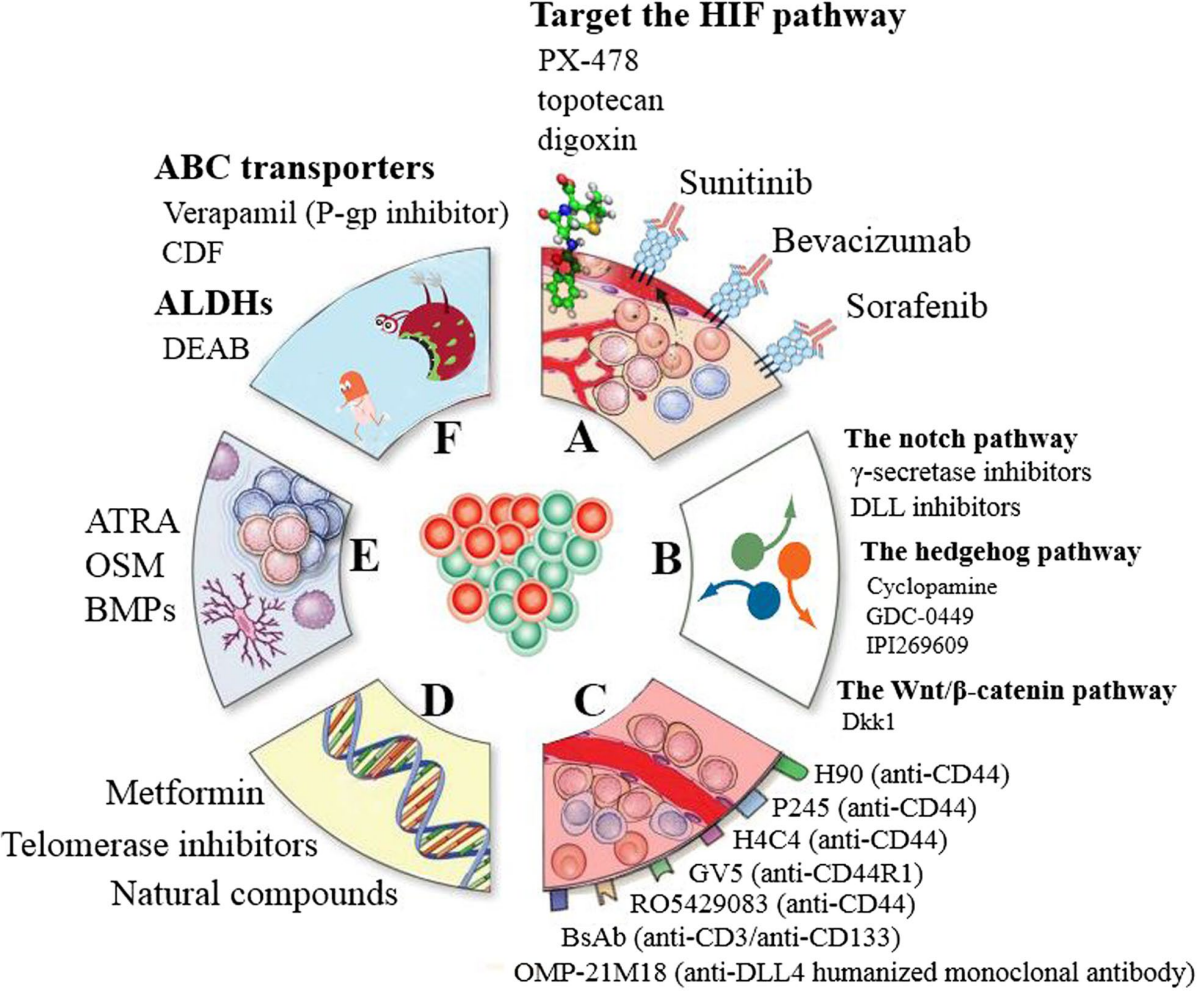
Cancer stem cells targeting**.** There are Different approaches for targeting cancer stem cells that can be used in a variety of cancers such as: (**A**) CSC niches: various types of cells and growth factors involving endothelial cells, immune cells, cancer associated fibroblasts (CAFs), various growth factors, and cytokines can be contained in the niche which provide a suitable microenvironment for tumor growth. Severe hypoxia and increased angiogenesis in the tumor microenvironment would cause a CSC niche to be formed near blood vessels. Along with these components, environment shifts, such as hypoxia, and pH have been introduced to contribute to the CSC niche. One of the important features of TME is low oxygen levels, referred to as hypoxia which turns out to maintain the stemness and thus malignancy of CSCs and finally promote tumor survival and metastasis. in response to hypoxia, the expression of the hypoxia-inducible factors (HIF1α, HIF-2α) are increased which can result in tumor malignancy. **B **Signaling pathways: One of the emerging targets for cancer treatment is the signaling pathways that regulate CSCs maintenance and survival. At present the Wnt, Notch, and Hh signaling pathways, as well as the TGF-β, JAK-STAT, PI3K, and NF-κB signaling pathways are the main signaling pathways which often interact with each other in CSCs during tumor development. Targeting the Wnt pathway has been proved to be difficult but noticeable progress has been made in early clinical trials of Notch and Hh pathway inhibitors. **C **Cell surface markers: targeting CSC surface markers is a potential CSC therapeutics approach and CD44 is one of the most commonly used and established CSC biomarkers which is a cell-surface extracellular matrix receptor. Many studies have introduced CD44 antibody therapy as the major anti-CSC approach. Another well-known CSC marker in several tumors such as glioblastoma, hepatocellular and colon cancers is CD133 which is a transmembrane glycoprotein. CD133 + CSCs have been proved to be resistant to chemotherapy and radiotherapy due to their lower proliferation, slower cell cycle, anti-apoptotic genes and higher expression of DNA repair. EpCAM has been discovered to be a CSC marker in solid tumors and is correlated with all CSCs characteristics. There is a significantly high frequency of tumor-initiating cells in EpCAM + /CD44 + /CD24− population in breast cancer. **D **Therapeutics molecules and (**E**) differentiation therapy: Metformin, salinomycin, DECA-14, rapamycin, Oncostatin M (OSM), some natural compounds, oncolytic viruses, microRNAs, signaling pathway inhibitors, TNF-related apoptosis inducing ligand (TRAIL), interferon (IFN), telomerase inhibitors, All-trans retinoic acid (ATRA) and monoclonal antibodies have recently been shown to suppress CSCs self-renewal in vitro and in vivo. A combination of these agents and conventional chemotherapy drugs can be used to dramatically hinder tumor growth, metastasis and recurrence; and (**F**) overcoming drug resistance in CSCs**:** Drug efflux leads to decreased intracellular drug concentration in CSCs through multi-drug resistance (MDR) transporters. Overexpression of ABCG2 which is one of the subfamilies of the ATP-binding cassette (ABCA-G) transporters is a major mechanism of chemoresistance in CSCs cells. The fourth generation of inhibitor drugs is in progress [4, 9–13]. *CSCs* cancer stem cells, *DLL* delta‑like ligand, *ATRA* all‑trans retinoic acid, *OSM* oncostatin M, *BMPs* bone morphogenetic proteins, *CDF* difluorinated curcumin, *ALDHs* aldehyde dehydrogenases, *DEAB* diethylaminobenzaldehyde, *HIF* hypoxia‑inducible factors

As mentioned, cancer stem cells (CSCs) induce metastasis and therapy resistance [5, 8, 9]. The functional or molecular properties relevant to CSC populations such as deregulation of pathways involved in differentiation, self-renewal, apoptosis and survival, increased expression of ATP binding cassette (ABC)-related transporters to efflux toxic compounds, adaptation to hypoxia, increased DNA damage response and reactive oxygen species (ROS) scavenging, altered metabolism, evasion of immunosurveillance and anchorage-independent survival and quiescence can mediate the acquired resistance [3, 8, 9]. The tumor microenvironment (TME), including cancer-associated fibroblasts (CAF) and extracellular matrix (ECM), play a key role in drug resistance [4, 10]. CSCs can be distinguished from the tumor-differentiated cells by evaluating the expression profiles of the surface markers classified in Table 2 based on tumor type. A number of CSCs targeted therapies which are proved to have the capability of preventing relapse have been depicted in Fig. 1.

**Table 2:** The common surface markers on Different CSCs

Malignancy	Surface marker	References
Brain	CD15 + , CD90^+^, CD133^+^, ABCG2^+^, CD49f^+^, CXCR4^+^, CD114^+^	[22, 29, 30]
Breast	CD133^+^, CD44^+a^, CD24^+^, EpCAM^+^, ALDH^high^, SSEA3^+^, SSEA4^+^, TRA-1–60, TRA-1–81^+^, TDGF1^+^, PODXL-1^+^, ABCG2^+^, CD10^+^, CXCR4^+^, CXCR1, 2^+^, CD55^+^	[31–34]
Colon	CD133^+^, CD44^+a^, CD24^+^, CD166^+^, EpCAM^+^, ALDH^high^, ESA^+^, TDGF1^+^	[28, 35, 36]
Endometr	CD44^+a^, EpCAM^+^, CD133^+^, ALDH^high^	[37, 38]
Gastric	CD133^+^, CD44^+a^, CD24^+^, CD54^+^, ALDH^high^, EpCAM^+^	[39, 40]
Hematological	CD19^+^, CD26^+^, CD34^+^, CD38^−^, CD123^+a^, PODXL-1^+^, TIM-3^+^, CD96^+^	[4, 41–43]
Head and Neck	CD271^+^, SSEA-1^+^, CD44^+a^, CD133^+^, CD10 +	[44, 45]
Liver	CD133^+^, CD44^+a^, CD49f^+^, CD90^+^, ALDH^high^, ABCG2^+^, CD24^+^, ESA^+^, EpCAM^+^, CD13^+^	[46–48]
Lung	CD133^+^, CD44^+a^, ALDH^high^, ABCG2^+^, CD87^+^, CD90^+^, SSEA1^+^, TDGF1^+^, PODXL-1^+^, Notch2^+^, CD56^+^	[49–51]
Melanoma	ABCB5^+^, CD20^+^, CD271 +	[52–54]
Pancreas	CD133^+^, CD44^+a^, CD24^+^, ALDH^high^, ABCG2^+^, EpCAM^+^, ESA^+^, PODXL-1^+^, Notch2^+^, CXCR4^+^, CXCR1, 2^+^	[55–57]
Prostate	CD133^+^, CD44^+a^, α2β1^+^, ALDH^high^, ABCG2^+^, TRA-1–60^+^	[58–60]
Testis	SSEA3^+^, SSEA4^+^, TRA-1-60^+^, TRA-1–81^+^, SSEA1^+^	[8, 61, 62]
Renal	SSEA1^+^, CD105^+^	[63, 64]
Ovary	CD133^+^, CD117^+^, DLL4^+^, CD44^+a^, CD24^+^, ALDH^high^	[65–67]
Colorectal	CD26^+^, LGR5^+^, DLL4^+^, CD44^+a^, CD133^+^, EpCAM^high^, ABCG2^+^, ALDH^high^	[68, 69]

^a^These markers expressed on both Cancer stem cells and normal tissue cells

Today, new therapies to overcome CSC resistance including epigenetic therapies, drugs targeting angiogenesis, immune cell-based therapies such as T, NK, and dendritic cells are rapidly developing, among which NK cells are of great interest because of their ability to identify and destroy cancer stem cells.

## NK cells as the ancient warriors against cancer

**Fig. 2:**
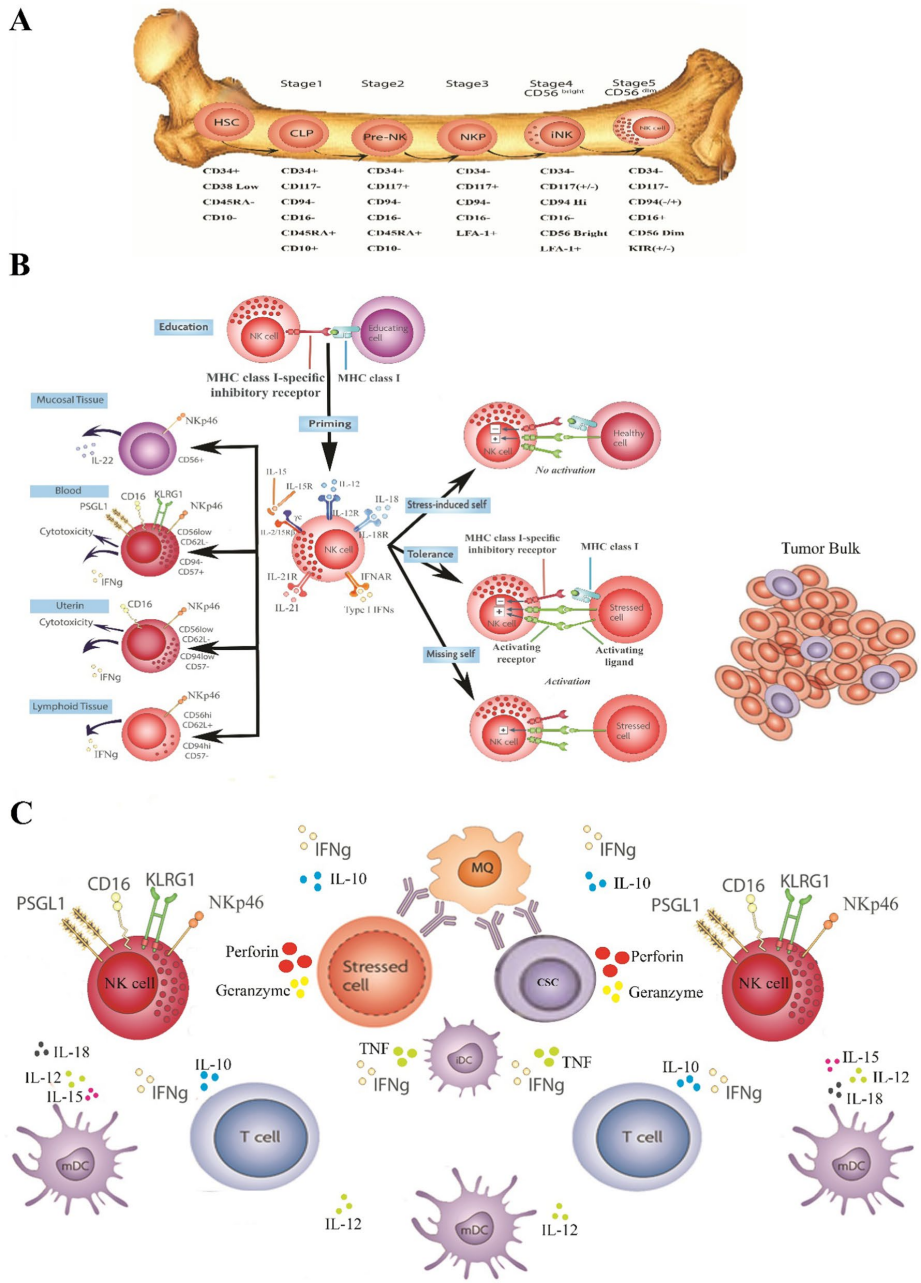
NK cell, from development to Functioning. **A** The pathway of NK cell generation and development; NK cells are derived from common lymphoid progenitor (CLP), and then enter the NK cell precursors (NKP) stage that express IL-7R and IL-2Rß 2Rß and IL-15 which play crucial roles in NK cells differentiation from CLPs to mature NK cells. These cells then express NKR-P, CD2, CD56, CD94 and KIRs and go through maturity and get ready for function. Mature NK cells also gain functional competence, expressing lytic molecules and cytokines such as Perforin, Granzyme A/B and IFN-γ. **B** NK cell educating and function; NK cells disappear from blood either by entering tissues, predominantly the spleen and the liver or through cell death. CD56^bright^ NK cells proliferate fast, but die relatively slowly which suggests that proliferating CD56^bright^ cells differentiate into CD56^dim^ NK cells in vivo. The peak of the effector NK cell expansion occurs at around 7–8 days after activation, regardless of the precursor frequency of antigen-specific NK cells, however it is difficult to detect memory NK after 4–5 months although they still exist. NK cells depict three mechanisms for their function on the target cells in 3 different pathways; 1. Missing-self in which the inhibitory receptors of MHC-I molecules are involved, and the cells are lysed in the down-regulation of MHC class I molecules, 2. Induced self-ligands in which NK cell activating receptors can detect stress molecules that are overexpressed by tumor cells, and ultimately lysing the target cell, 3. Antibody-dependent NK cell-mediated cytotoxicity in which specific antibodies of tumor antigens are binding to CD16 and subsequently cause the target cell to lyse (21, 22). **C** NK cells can play modulatory role in the immune system against tumor and infected cells, and affect T cells and macrophages and prepare them to serve, and also have a significant contribution to the process of maturation of dendritic cells. *HSCs* hematopoietic stem cells; mNK cell, mature NK cell

The peripheral blood NK cells are divided into two subsets of CD56^bright^ and CD56^dim^ during the developing stages and based on the expression of this receptor, and after education and maturation (Fig. 2A, B), they are distributed into different organs (Fig. 2B) based on their unique characteristics [16]. CD56^bright^ CD16^neg^ NKG2A^+^ KIR^neg^ NK cells are more immature than CD56^dim^ NKG2A^±^ KIR^+^ CD16^+^ NK cells. Moreover, CD56^dim^ NKG2A^±^ KIR^+^ CD16^+^ NK cell subset includes more mature NK cell subset represented by CD57^+^ KIR^+^ NKG2A^−^ NKG2C^+^ adaptive NK cells [17, 18]. IL-2, IL-4, IL-7, IL-10, IL-12, IL-18, IL-21, type I and type II IFN, and TGF-ß are the cytokines which play essential roles in NK cells maturation (Fig. 2B) [19–21]. The gun-house of NK cells is packed with granular weapons called Perforin and Granzyme which are released in the synaptic space between NK cells and their target, destroying the target cells (Fig. 2B, C) [22]. Due to their immunological mediating role, these cells form a desirable connection between the innate and adaptive immunity responses to cancer cells (Fig. 2C) [23, 24]. NK cell capability to destroy and eliminate the target cells depends on the balance of its activating and inhibitory signals [25, 26] i.e. the ligands expressed on the target cells interact with the NK cell surface activating and inhibitory receptors and trigger the activating or inhibitory signals, so NK cells are not controlled by antigen specificity [26–29].

NK cells functional mechanism is based on the identification of MHC class I (HLA class I) molecules as ligands for NK cell receptor group of killer cell immunoglobulin-like receptors (KIRs) that are able to bind four types of MHC class I in human (HLA-A, HLA-B, HLA-C and HLA-G). MHC class I molecules expressed on the surface of healthy cells can act as inhibitory ligands in binding to their receptors on the surface of NK cells and as a result cause the self-tolerance of NK cells. The expression of MHC molecules in abnormal and tumor cells is decreased which reduces the induction of inhibitory signals in NK cells. As a result, the balance of signals shifts to NK cell activation and elimination of target cells.

Moreover, cancer cells, especially CSCs, express low/no levels of MHC class I (the missing self-hypothesis), so they are highly susceptible to destruction by NK cells [9]. Some CSCs, in addition to low MHC class I expression levels, express high levels of NK cell activating markers and are therefore more susceptible to be killed by NK cells [30–32]. The expression of several CSC markers (Table 2) such as CD24, CD44, CD133 and ALDH can increase the elimination susceptibility of these cells by the activated NK cells through stimulation of NK activation markers such as MICA/B, Fas and Death receptors [8, 11, 32, 33].

The previous studies on melanoma, colorectal and glioblastoma have shown that NK cells are more likely to target CSCs than non-CSCs in heterogeneous solid tumor populations without any pharmacological pretreatment [30, 31, 76]. Another study on CD44 + CD24− human breast CSCs demonstrated that these cells were sensitive to NK cells activated by Interleukin 15 and 2, and these effects were due to the increased expression of NKG2D ligands, ULBP1, ULBP2 and MICA, on these CSCs [77, 78]. CSCs having low MHC-I and beta microglobulin levels can facilitate the escape from immune system attack, considering that downmodulation of MHC-I expression on CSCs can facilitate the escape from cytotoxic T lymphocyte-mediated immune responses. CTL-mediated killing is based on self-MHC molecule that is expressed on the antigen-specific target cells that must be recognized by T cell receptor (TCR), therefore the histocompatibility complex (MHC) restriction is a major limitation of this process. On the contrary, NK cells can kill tumor cells that do not express HLA-I molecules but NK cell therapy has its own challenges as CSCs can escape from NK cells killing response via some alternative pathways which are mentioned accordingly [36, 79].

A study on the immunogenicity of CD133 + brain tumor stem cells (BTSCs) has shown a downregulation in MHC class-I (MHC I) expression or NK cell activating ligands on the majority of CD133 + cells, which may make these cells resistant to the adaptive and innate immune surveillance [79]. However, both CD133− and CD133 + cells of melanoma, were vulnerable to IL-2 activated allogenic NK cells and responded to the DNAM-1 ligands Nestin-2 and PVR [80]. High levels of anti-apoptotic proteins such as Bcl-2, Bcl-xL and surviving proteins protect CSCs from NK cells and cytotoxic T cells responses [81]. The data obtained from another study focusing on MICA and MICB (MHC class I‐related chain A and B) in TME (tumor microenvironment), revealed that these stimulatory NKG2D receptor ligands are downregulated due to the aberrant expression of oncogenic miR‐20a in human breast CSCs and ultimately lead to the escape of these CSCs from NK cell killing [28, 82, 83]. Breast CSCs having a CD44^high^/CD24^low^ phenotype can also escape the effect of NK cells by eliciting resistance to trastuzumab-mediated antibody-dependent cell-mediated cytotoxicity (ADCC) [83]. Colon CSCs secrete high levels of IL-4 which is a cytokine promoting drug resistance and inhibiting the immune response to tumors [31, 42]. Some CSCs associated with certain types of cancer resist the NK cells killing by not expressing NK cell activating ligands such as NKG2D, NKp30 and NKp44 and also increasing the expression of inhibitory ligands such as CD94/NKG2A [79, 83]. The previous studies have shown that these expression changes in surface markers and ligands associated with NK cell receptors can reduce the expression level of CD16 during NK cells development and consequently reduce the cytotoxic ability by interaction with CSCs. In this phase, NK cells are in a state called “split anergy” in which only the production of interferon-gamma (IFN-γ) and tumor necrosis factor-alpha (TNF-α) is maintained [33, 84, 85]. This functional state is critical for tumor differentiation and the functional NK cell inactivation [33, 86, 87]. It is important to note that the activity of NK cells is inhibited by the immunosuppressive factors such as TGF-β, IL-6, IL-8, IFN-γ, MICA,B released in TME [88] and the differences between the CSCs and other tumor cells secretomes may also inhibit cytotoxic NK cells more strongly [8].

To develop NK cell-based therapies, it is important to find appropriate sources of these cells that minimize cell number limitations, immunological complications, and HLA Donor-Recipient matching [89–91]. There are several sources of NK cell including peripheral blood (PB), umbilical cord blood (UCB) [92], bone marrow (BM) and cell lines [93]. Deriving NK cells from PBMC, hESC and iPSC sources was a revolution which wiped out the worries about the low number and purity of NK cells forever [94]. The use of PBMC, hESC and iPSC sources as homogeneous and off-the-shelf sources for NK cells would open up the possibility to create clinical cell banks available to patients on demand and these features can also provide an unlimited source of genetically engineered NK cell products and the emergence of new tool called CAR-NK for cancer treatment [95–98]. The effects of different types of NK cell sources in clinical evaluation for metastatic cancers have been compared and the results are depicted in Table 3.

**Table 3:** Comparison of the effects of different types of NK cell immunotherapies for targeting malignancies

NK source	Advantages	Disadvantages
Autologous NK cells	Universal Safe	Low efficacy
Allogenic NK cells	Highly effective against some malignancies	no standard protocols or products
CAR NK cells	Highly potentiate NK cell antitumor activity; more efficiency and safer than CAR T cells	Difficult manipulate Difficult expansion
NK cell lines	Unlimited Homogeneous well-defined highly active population low cost	Low efficacy safety concerns need to be irradiated

Therefore, the mechanisms described can be used to target cancer stem cells and provide alternative approaches to design CAR-NK cells so as to produce desirable NK cells that promote optimal cytotoxicity against cancer stem cells as well as being highly resistant to inactivation mechanisms.

## CAR NK cell as a novel tool to target CSCs

The design and use of CARs to reach PBMC, hESC and iPSC- derived NK cells is based on genetic engineering and modifying and using a variety of cytokines, growth factors, feeder’s layer and monoclonal antibodies [95]. CAR-immune effector cells have the ability to recognize the tumor associated antigens (TAAs), and subsequently eliminate the target cells through inducing the cytotoxic factors such as perforin and granzyme [99]. To date, five generations of CAR have been introduced, and its construct is based on three regions, including the extracellular domain consisting of a single-chain variable fragment (scFv), a linker that is flexible and attached through a spacer to the transmembrane domain and an intracellular signaling domain of immunoreceptor tyrosine-based activation motifs (ITAM) TCR or a cytoplasmic domain of other activating receptors. The first generation of CARs consisted of a single-chain variable fragment (scFv) recognizing the tumor surface antigens, and an immunoreceptor tyrosine-based activation motif (ITAM) which is usually CD3ζ chain as an intracellular signaling domain [100]. The second and third generations of CAR platforms are being used According to the dual-signal model activation. These structures introduced CD28, CD134 (OX40), CD137 (4-1BB) and 2B4 and other costimulatory molecules, pursuing the goal of increasing cytotoxicity [101]. The fourth and fifth generations are also based on the second generation but replacing the additional costimulatory molecule of the third generation with protein inducers (IL-12, IL-18) and IL-2 receptor β-chain domain in the fourth generation and STAT3 binding site in the fifth generation to overcome the immunosuppressive tumor microenvironment. Therefore, these approaches provide the three signals that are required for CAR-modified cell activation [102]. Despite obtaining successful results in CAR-T cell therapy against tumor cells, CAR-NK cells offer many advantages for cancer immunotherapy [103] which are mentioned in Table 4.

**Table 4:** The comparison of CAR-T cells with CAR-NK cells

	CAR-NK	CAR-T
Source	Various	Limited
Expression of surface receptor (Ag-specific receptor)	Not required (germ line-encode)	Required (rearranged Ag-specific)
Prior sensitization	Not required	Required
Collection	Leukopheresis	Leukopheresis
Preparation	Autologous: CD56^+^ Enrichment Allogeneic: MHC-matched donor selection or alloreactive T-cells depletion	Activation of cells with anti-CD3/CD28 beads Allogeneic donor: MHC match required
Expansion	engineered feeders required (example: K562 cells expressing IL-15 and TNFSF9) plus IL-2 (in flasks, bags or bioreactors)	Flasks, bags or wave expansion system
Transduction	Low transfection efficiency even with viral vectors	Desirable transfection efficacy Ex: Lentiviral systems transduce about 1/3 of T cells
Cytotoxic mechanisms	Multiple receptors can trigger CAR-independent and FcR-dependent cytotoxicity	CAR-restricted killing In case of antigen loss on tumors, CAR-expressing T cells become ineffective
Escaped tumor and infected cells recognition	Yes	No
Clinical results	Proof of clinical benefit pending	Phase II studies have shown clinical benefit
In vivo functionality	No need for suicide gene	Suicide genes are required to control life span in vivo
HLA expression-related recognition	Dependent	Independent
GVHD	Low/no	High/yes
Cytokine-induced killer cells	No	Yes
Toxicity	Low	High (neurotoxicity)
Safety	High/low safe	Low/no safe
Side effects	Limited life span in patients	“off target” effect prolonged Survival period in patient’s circulation CRS MQ activation syndrome Hemophagocytic Lymphohistiocytosis (hlh)
Off-the-shelf availability	Present	Missing (preparation required for each patient)
Cost	Cost benefit	Expensive

One of the most significant advantage of NK cells is the limited lifespan after activation which eliminates the need for the presence of the suicide gene as a safety key in the CAR structure [104].

As of April 2021, twenty-three studies have been submitted to Clintrials.gov to evaluate the safety and efficacy of CAR-NK cells in cancer patients (Table 5).

**Table 5:** CAR-NK cells in clinical trials

Tumor type	Condition or disease	Origin of NK cell	Target	Status	Phase	Country	Clinical trial ID
Hematological malignancy	B-ALL	Haploidentical PB-NK	CD19	Recruiting	II	Singapore	NCT01974479
	B-ALL	Haploidentical PB-NK	CD19	Completed	I	USA	NCT00995137
	Lymphoma and leukaemia	NK-92	CD7	Recruiting	I/II	China	NCT02742727
	Lymphoma and leukaemia	NK-92	CD19	Recruiting	I/II	China	NCT02892695
	Refractory B-cell lymphoma	Unknown	CD19	Not recruiting	Early I	China	NCT03690310
	Relapsed or Refractory B Cell Non-Hodgkin Lymphoma	Unknown	CD19	Not recruiting	Early Phase 1	China	NCT04639739
	Relapsed and refractory B cell malignancies	Unknown		Recruiting	I/II	China	NCT04747093
	Refractory B-cell lymphoma	Unknown	CD19/CD22	not recruiting	Early I	China	NCT03824964
	AML	NK-92	CD33	Completed	I/II	China	NCT02944162
	Lymphoma and leukaemia (relapsed/refractory B-cell malignancy)	Umbilical cord blood	CD19	Recruiting	I/II	USA	NCT03056339
	Refractory B-cell lymphoma	Unknown	CD22	Not recruiting	Early I	China	NCT03692767
	Lymphoma and leukaemia	Umbilical cord blood	CD19	Withdrawn	I/II	USA	NCT03579927
	relapsed/refractory multiple myeloma	NK92	BCMA	Recruiting	I/II	China	NCT03940833
	B-cell lymphoma, CLL	iPSC (FT596)	CD19	Recruiting	I	USA	NCT04245722
Solid tumor	Metastatic solid tumor	PB-NK	NKG2DL	Recruiting	I	China	NCT03415100
	Glioblastoma	NK-92	HER2	Recruiting	I	Germany	NCT03383978
	Non-small cell lung cancer	NK-92	–	Recruiting	I	China	NCT03656705
	Solid tumor	NK-92	ROBO1	Recruiting	I/II	China	NCT03931720
	Solid tumors	NK-92	ROBO1	Recruiting	I/II	China	NCT03940820
	Pancreatic cancer	NK-92	ROBO1	Recruiting	I/II	China	NCT03941457
	Epithelial ovarian cancer	PB-NK	Mesothelin	not recruiting	Early I	China	NCT03692637
	Castration-resistant prostate cancer/	PB-NK	PSMA	Not recruiting	Early I	China	NCT03692663
	Solid tumor	NK-92	MUCI	Recruiting	I/II	China	NCT02839954

**Fig. 3:**
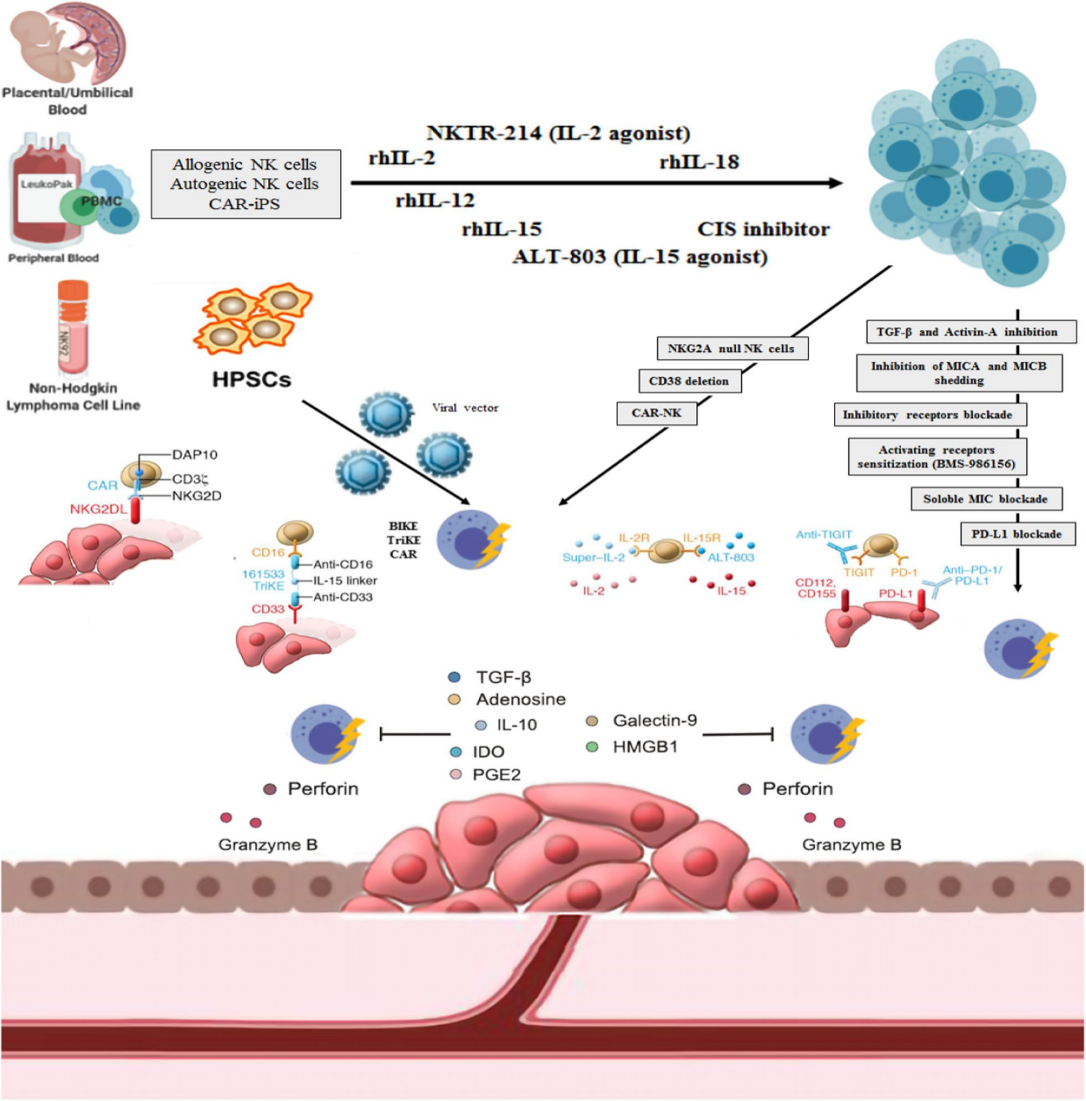
Strategies for achieving functional NK cells against tumor cells

The studies mentioned in Table 5 used the approaches of overcoming the major challenges of achieving functional NK cells against tumors, especially the solid tumors (Fig. 3). These challenges are classified into the following groups:

## Providing NK cells

There are still many potential challenges in providing NK cells from the desirable sources as well as maintaining the NK cell expansion in the recipients’ body. Allogeneic NK cells are widely used in clinical trials today, however, after development which has the possibility of infusion contamination with T or B cells in the expanded NK cell preparation, undesirable immune responses can be induced in the recipient, such as GVHD or post-transplant lymphoproliferative disease [93]. As mentioned, NK cells have a short life after gaining their killing function. Therefore, maintaining the number of functional NK cells is another challenge in immunotherapy with NK cells.

The use of CAR-NK cells expressing IL-2 and IL-15 is suggested as an applied strategy for overcoming the mentioned challenges. Liu et al. used a single infusion of CD19-CAR/IL15 + CB-NK cells in Raji lymphoma mouse models with the approach of increasing the function and survival of NK cells which was successful in controlling the tumor progression [105]. MD Anderson Cancer Center has run a dose escalation study phase I/II of umbilical cord blood-derived CAR-engineered NK cells in conjunction with lymphodepleting chemotherapy in Relapsed/Refractory B-Lymphoid malignancies (NCT03056339) with the aim of investigating the highest tolerable dose of CAR-NK cells for the patients with relapsed or refractory B-cell lymphoma or leukemia. In this study, CAR-NK (iC9/CAR.19/IL15-transduced CB-NK cells) cells were used after chemotherapy, as a result of which some improvements were observed in the disease in stem cell transplant patients with relapsed or refractory (has not responded to treatment) B-cell lymphoma or leukemia. A pilot study of NKG2D-ligand targeting CAR-NK cells in metastatic solid tumors patients is ongoing in its first phase in Guangzhou China in which some of patients are going to receive Interleukin-2 (IL-2) subcutaneously following the infusion of CAR-NK cells to support the in vivo survival of CAR-NK cells (NCT03415100). Another study is going to use engineered NK-92 as specific CAR-pNK cell to target relapsed/refractory CD33 + AML in which NK92 cells are engineered to contain anti-CD33 attached to TCRζ, CD28 and 4-1BB signaling domains (NCT02944162). In another ongoing study anti-CD19 attached to TCRζ, CD28 and 4-1BB signaling domains is being used for generating allogenic CAR-NK cells in treating patients with CD19 positive relapsed or refractory leukemia and lymphoma (NCT02892695).

### NK trafficking and infiltrating into tumor sites

Migration and localization of NK cells in the tumor sites and their number infiltrated into tumor is another major challenge for CAR-NK therapy. To overcome the anatomical barriers, different injection approaches such as local injection, intra-peritoneal injection, and focused ultrasound-guided delivery can be used [106]. HER2 has been shown to be up-regulated in several solid tumors including brain, breast, colon, and ovary making it a desirable target for CAR-NK cells therapy [96–98]. In a preclinical study, anti-HER2 CAR-NK-92 cells were delivered into the brain of mice with metastatic breast cancer using focused ultrasound [107].

To increase the infiltration and homing capacity, CARs can be used that express chemokine receptors (CCRs) compatible with chemokine CC (CCL) ligands of tumor cells, for example, CXCR2-expressing NK cells show significant migration to tumors expressing CXCR2 ligands [108, 109]. In the treatment of CXCL12/SDF-1α-secreting glioblastoma cells, anti-EGFRvIII CAR NK cells were used with overexpression of CXCR4 and the results showed an increase in chemotaxis towards these tumor cells, complete tumor remission in a number of mice, and an increased overall survival [110].

Modification of TME by oncolytic viruses can induce an inflammatory immune response, followed by an enhanced immune cell trafficking [111]. In a preclinical study, the combination of EGFR-CAR NK-92 cells with oHSV-1 in mice with breast cancer tumor cells in the brain resulted in more efficient removal of the tumor cells in the brain as well as the increased survival [112].

The use of specific markers of a particular type of cancer (mentioned in Table 2) is also one of the approaches that have been considered in some studies today among which we can mention NCT03692637 study, targeting epithelial ovarian cancer by anti-mesothelin CAR-NK cells and another study (NCT03692663) using anti-PSMA CAR-NK cells in patients with castration-resistant prostate cancer.

### Lack of NK function in the tumor microenvironment

Tumor cells recruit neutrophils, macrophages, Tregs and immunosuppressive myeloid suppressor cells (MDSCs) in TME which produce factors such as TGF-β, IL-10, PD-1 and arginase to escape the immune responses providing a strong immunosuppressive environment for tumor growth [113]. Several strategies have been reported to preserve the function of NK cells in vivo and minimize the effects of the mentioned inhibitory factors. For example, the use of TGF-β kinase inhibitors with NK cells can maintain the potential for cytotoxicity and expression of NKG2D and CD16 activating receptors [114]. To increase the antitumor activity in NK-92 cells, a hybrid CARs with an extracellular TGF-β receptor domain linked to an intracellular NKG2D domain as well as knocking down SMAD3 was used which lead to improving the function of NK cells against the solid tumors [115, 116]. Also, to increase the cytotoxicity of NK cells against tumor cells, a narrow-spectrum histone deacetylase inhibitor called Entinostat was used, which increased MICA expression on tumor cells and NKG2D expression in primary NK cells, even in the hypoxic environment [117]. NK cells in hypoxic environment would face a downregulated expression of activating receptors such as NKp30, NKp46, NKp44 and NKG2D, and a suitable environment is provided for tumor progression due to inducing metabolic disturbance, increasing angiogenesis and expression of tumor growth factors [13, 113].

CD73 induces the expression of arginase under hypoxic conditions and inhibits NK cell functions. In a preclinical study on mouse models with lung cancer, CD73 inhibition was used in combination with NKG2D-CAR-NK. CD73 inhibition in the tumor cells expressing NKG2D caused an increased NKG2D-CAR-NK cells infiltration into the tumor site and an improved anti-tumor response [118].

The expression of checkpoint proteins on tumor cells such as PD-1, CTLA-4, LAG3, and TIGIT can provide immune surveillance evasion. Therefore, the use of checkpoint protein blockers in the CARs structure can provide the possibility of an improved cytotoxic function [119]. The combination of PD-L1-CAR-NK-92 cells with high-affinity CD16 and IL-2 has high levels of perforin and granzyme expression against human cancer cell lines including breast, lung, and gastric cancers [119–124].

## The efficacy of CARs transduction methods

Today, two approaches are used for the transduction of CARs construct including viral and non-viral based methods. The efficacy of transduction methods is one of the challenges to be considered in using CAR-modified NK cells [125]. viral vectors (Lentivirus and Retrovirus) applied for CAR transduction have high potential and efficacy in the clinical uses while having some limitations such as the risk of inducing mutagenesis, which is not desirable for human clinical use [100, 125, 126]. The use of non-viral methods such as mRNA electroporation, also has its own limitations such as the lack of integration of transcripts into the genome because of its short-expression time [127]. The quantity of mRNA used are also critical in clinical applications which should be compensated by multiple infusions for the transient expression of CAR proteins [127, 128]. The efficiency of viral and non-viral transduction methods has been compared with each other in Table 6.

**Table 6:** comparison of the efficiency of Viral and non-viral transduction methods

Source of NK cells		Transduction method	Transduction vector	Transduction efficiency (%)	References
Primary cells		Viral-based	Lentivirus	16–80	[111, 118]
			retrovirus	27–75	[119]
		Non-viral based	mRNA transfection	10–85	[120, 121]
			Trogocytosis	24–47	[122]
Cell lines	NK-92	Viral-based	Lentivirus	15–26	[121, 123, 124]
		Non-viral based	mRNA transfection	56	[121]
			*PiggyBac*	84	[111]
	YTS	Viral-based	Lentivirus	30–98	[111]
	LNK			30–40	[124]
	DERL7			30–40	
UCB-derived Cells		Viral-based	Lentivirus	12–73	[121]
			retrovirus	49–67	[125, 126]
iPSCs		Non-viral based	pKT2-mCAG-IRES	–	[127, 128]
			*PiggyBac*		

## Tumor antigen heterogeneity

Finding a target antigen on the surface of a tumor cell that is very uniformly expressed is the most important step in designing CARs. Clonal evolution and downregulation of TAA expression can cause significant differences in TAA expression between the single-cell clones, allowing tumors to evade the immune surveillance. Most TAAs are expressed by cells not only in tumor especially solid tumors, but also in vital organs, making it impossible to avoid “on-target, off-tumor” effects [100, 113, 119, 137]. Using bispecific CARs which have the ability to recognize two different antigens and are activated by the binding of both antigens provides the possibility of relative overcoming of this problem [100, 138, 139]. BCMA-CAR-NK 92 cells are being used in the relapsed and refractory multiple myeloma patients with BCMA (B-cell maturation antigen) expression in Suzhou Hospital Affiliated to Nanjing Medical University with the purpose of enabling NK-92 cells by CARs to recognize and kill MM cells through targeting BCMA (NCT03940833). Another study in phase 1 and 2 of clinical trials is also being conducted by the same group focusing on the anti-tumor responses of BiCAR-NK/T cells on patients with solid tumors without any conditioning chemotherapeutic regimen (NCT03940820). Three studies focusing on targeting ROBO1 by BiCAR-NK/T cells (ROBO1-CAR-NK/T cells) are ongoing in China and are intended to investigate the effects of CAR-NK cells on pancreatic cancer (NCT03941457), solid (NCT03940820) and malignant (NCT03931720) tumors.

Studies on targeting cancer stem cells in the field of research are numerous, but limited in clinical trials. Therefore, it seems that the development of the novel therapeutic modalities such as the combination of CAR engineering with CRISPR-Cas9 gene editing in primary NK cells may lead to successful clinical trials.

## Conclusion

NK cells with their specific characteristics against tumor cells have opened up a new world in treating and fighting against cancer [140]. These cells have shown remarkable anti-tumor function against hematologic cancers, but their function against solid tumors has remained unclear [141]. However, the significant point about these cells is that they can be used in many advanced malignancies for adoptive cell therapy [4, 11, 140]. CAR technology in this scenario, has been proved to be very helpful. Through this technology, the resulting NK engineered cells can both recognize tumor cells and use their killing ability against them. In addition, if we arm the engineered cells against cancer stem cells, we can succeed in removing them. The NK cells, as warriors in the cancer cells dark world, have emerged and attracted much attention and that is why many studies on cancer NK cell therapy are ongoing today.

## Future perspective

Recent advances in gene editing technology have let us imagine its potential applications for the creation of novel CAR-NK cells with anti-tumor activity having limited cytotoxicity to normal tissues in clinical trials. The novel strategies, such as CRISPR-Cas9 genetic modifications as innovative methods can introduce alternative platforms for overcoming the current limitations of NK cell-based therapy including the fact that the positive signal induced by CAR is only partially inhibited by the negative signal generated by KIRs or NKG2A in allogenic transplantations.

## Data Availability

No applicable.
